# Supplementary material for: Large-scale GWAS of food liking reveals genetic determinants and genetic correlations with distinct neurophysiological traits
Source: Nat Commun. 2022 May 18;13:2743. doi: 10.1038/s41467-022-30187-w (PMC9117208; doi:10.1038/s41467-022-30187-w)
Supplement: Supplementary file 3 — Description of Additional Supplementary Files [file 41467_2022_30187_MOESM3_ESM.pdf]

Description of Additional Supplementary Files

File Name: Supplementary Data 1
Description: Summary statistics of the food liking traits.

File Name: Supplementary Data 2
Description: Cohort descriptors and information.

File Name: Supplementary Data 3
Description: List of loci used for the enrichment analysis.

File Name: Supplementary Data 4
Description: MRI genetic correlations.

File Name: Supplementary Data 5
Description: Food liking and consumption genetic correlation and heritability.

File Name: Supplementary Data 6
Description: Significant associations. The table reports the summary statistics of each significant association, only the top SNP per trait per locus is reported.

File Name: Supplementary Data 7
Description: Hypercoloc clustering.

File Name: Supplementary Data 8
Description: Number of hypercoloc clusters.

File Name: Supplementary Data 9
Description: Replication SNPs.

File Name: Supplementary Data 10
Description: Conditioned analysis results.

File Name: Supplementary Data 11
Description: FUMA tissue enrichment analysis.

File Name: Supplementary Data 12
Description: Go term Enrichment.

File Name: Supplementary Data 13
Description: Genetic correlations between the food-liking and brain MRI traits.

File Name: Supplementary Data 14
Description: Genetic correlation between liking and consumption of the same foods.
