# Supplementary material for: Large-scale GWAS of food liking reveals genetic determinants and genetic correlations with distinct neurophysiological traits
Source: Nat Commun. 2022 May 18;13:2743. doi: 10.1038/s41467-022-30187-w (PMC9117208; doi:10.1038/s41467-022-30187-w)
Supplement: Supplementary file 5 — Reporting Summary [file 41467_2022_30187_MOESM5_ESM.pdf]

Corresponding author(s): Nicola Pirastu
Last updated by author(s): Mar 30, 2022

Reporting Summary

Nature Portfolio wishes to improve the reproducibility of the work that we publish. This form provides structure for consistency and transparency in reporting. For further information on Nature Portfolio policies, see our Editorial Policies and the Editorial Policy Checklist.

Statistics

For all statistical analyses, confirm that the following items are present in the figure legend, table legend, main text, or Methods section.

n/a	Confirmed	
☐	☒	The exact sample size (*n*) for each experimental group/condition, given as a discrete number and unit of measurement
☐	☒	A statement on whether measurements were taken from distinct samples or whether the same sample was measured repeatedly
☐	☒	The statistical test(s) used AND whether they are one- or two-sided<br>*Only common tests should be described solely by name; describe more complex techniques in the Methods section.*
☐	☒	A description of all covariates tested
☐	☒	A description of any assumptions or corrections, such as tests of normality and adjustment for multiple comparisons
☐	☒	A full description of the statistical parameters including central tendency (e.g. means) or other basic estimates (e.g. regression coefficient) AND variation (e.g. standard deviation) or associated estimates of uncertainty (e.g. confidence intervals)
☐	☒	For null hypothesis testing, the test statistic (e.g. *F*, *t*, *r*) with confidence intervals, effect sizes, degrees of freedom and *P* value noted<br>*Give P values as exact values whenever suitable.*
☒	☐	For Bayesian analysis, information on the choice of priors and Markov chain Monte Carlo settings
☐	☒	For hierarchical and complex designs, identification of the appropriate level for tests and full reporting of outcomes
☐	☒	Estimates of effect sizes (e.g. Cohen's *d*, Pearson's *r*), indicating how they were calculated

Our web collection on statistics for biologists contains articles on many of the points above.

Software and code

Policy information about availability of computer code

Data collection	Data was collected by the UK biobank team through an online questionnaire. Detailed description of the questionnaire and the questionnaire itself can be found here: https://biobank.ctsu.ox.ac.uk/crystal/ukb/docs/foodpref.pdf<br>Example code can be found here: https://osf.io/e43x5/
Data analysis	All software used is available and detailed in the manuscript methods section.<br><br>Software tools used:<br>ldsc (v1.0.1)<br>ldhub<br><br>R (v. 3.6.1) packages used:<br>GenomicSEM (0.0.5c)<br>Stats (v 3.6.2)<br>Clusterprofiler (3.16.1)

For manuscripts utilizing custom algorithms or software that are central to the research but not yet described in published literature, software must be made available to editors and reviewers. We strongly encourage code deposition in a community repository (e.g. GitHub). See the Nature Portfolio guidelines for submitting code & software for further information.

Data

Policy information about availability of data

All manuscripts must include a data availability statement. This statement should provide the following information, where applicable:

- Accession codes, unique identifiers, or web links for publicly available datasets
- A description of any restrictions on data availability
- For clinical datasets or third party data, please ensure that the statement adheres to our policy

All GWAS results will be available through GWAS catalog at the time of publication.
All GWAS results have been made available through GWAS catalogue accession number GCP000266
Supplementary files 1-3 can be downloaded at: https://osf.io/e43x5/

Field-specific reporting

Please select the one below that is the best fit for your research. If you are not sure, read the appropriate sections before making your selection.

☒ Life sciences ☐ Behavioural & social sciences ☐ Ecological, evolutionary & environmental sciences

For a reference copy of the document with all sections, see nature.com/documents/nr-reporting-summary-flat.pdf

Life sciences study design

All studies must disclose on these points even when the disclosure is negative.

Sample size	Sample size for this study was up to 165,625 subjects for the discovery GWAS. Sample size was chosen based on the number of individuals in UKBB who had completed the food-liking questionnaire. With >160k full responses this makes it the most powered study of the genetics of food-liking to date.
Data exclusions	Only subjects who completed the food liking questionnaire and were of European descent were included in the study.
Replication	Replication was sought in up to 26,154 collected in 11 different cohorts and was carried out for all those SNP trait associations which were available in at least 10 thousand people. Although replication was broadly successful, this was not the always the case . Despite our attempts to replicate many of the hundreds of food-liking associations, due to varying sample sizes, we were underpowered to be completely successful.
Randomization	Randomization was not applicable to the study as there was no data being analysed which would allow identification of any individuals in the study.
Blinding	Blinding is not necessary or applicable in GWAS analyses.

Reporting for specific materials, systems and methods

We require information from authors about some types of materials, experimental systems and methods used in many studies. Here, indicate whether each material, system or method listed is relevant to your study. If you are not sure if a list item applies to your research, read the appropriate section before selecting a response.

Materials & experimental systems

n/a	Involved in the study	
☒	☐	Antibodies
☒	☐	Eukaryotic cell lines
☒	☐	Palaeontology and archaeology
☒	☐	Animals and other organisms
☐	☒	Human research participants
☒	☐	Clinical data
☒	☐	Dual use research of concern

Methods

n/a	Involved in the study	
☒	☐	ChIP-seq
☒	☐	Flow cytometry
☒	☐	MRI-based neuroimaging

Human research participants

Policy information about studies involving human research participants

Population characteristics	The UK Biobank cohort is a population-based cohort of approximately 500,000 participants that were recruited in the United Kingdom between 2006 and 20106 . Invitations to participate were sent out to approximately 9.2 million individuals aged between 40 and 69 who lived within 25 miles of one of the 22 assessment centers in England, Wales, and Scotland. The participation rate for the baseline assessment was about 5.5%.<br>Details for each replication cohort can be found in Supplementary table S2<br>For the GWAS covariates used included batch number, a genetic relationship matrix and genotyping array. Gender was also used and ~57% of participants were female.
Recruitment	Individuals were recruited by e-mailing all current participants of UKBB and requesting the fill in the questionnaire. This comes with a number of potential biases, particularly that members of UKBB tend to be older, more highly educated and wealthier than the general UK population. This is also particularly true of those who then return to UKBB secondary analyses. Details of recruitment are reported in the Materials and Methods section.
Ethics oversight	UK Biobank was approved by the North West Multi-Centre Research Ethics Committee (MREC) and in Scotland, UK Biobank was approved by the Community Health Index Advisory Group (CHIAG).<br>Each cohort obtained ethical approval from the relevant Institution according to each country legislation.

Note that full information on the approval of the study protocol must also be provided in the manuscript.
